# A GH42 β‑Galactosidase from the Human Isolate *Bifidobacterium breve* DSM 20213: Biochemical and Transgalactosylation Properties Reveal the Potential for Galacto-oligosaccharides Synthesis

**DOI:** 10.1021/acsomega.5c06811

**Published:** 2025-12-17

**Authors:** Khanh-Trang Vu-Le, Konlarat Phirom-on, Sineenat Sripattanakul, Dinh Binh Chu, Stephan Hann, Markus Blaukopf, Leander Suetzl, Chris Oostenbrink, Dietmar Haltrich, Thu-Ha Nguyen

**Affiliations:** 1 Food Biotechnology Laboratory, Department of Biotechnology and Food Science, 27270 BOKU University, Muthgasse 18, Vienna A-1190, Austria; 2 Faculty of Biology and Environmental Science, The University of Danang-University of Science and Education, Danang 550000, Vietnam; 3 Office of Research Administration, Chiang Mai University, Chiang Mai 50200, Thailand; 4 Laboratory of BioMolecular Imaging, Molecular and Cellular Biology, Department of Radiologic Technology, Faculty of Associated Medical Sciences, Chiang Mai University, Chiang Mai 50200, Thailand; 5 Faculty of Chemistry, School of Chemistry and Life Sciences, 118018 Hanoi University of Science and Technology, 1 Dai Co Viet, Hanoi 100000, Vietnam; 6 Institute of Analytical Chemistry, Department of Natural Sciences and Sustainable Resources, 27270 BOKU University, Muthgasse 18, Vienna A-1190, Austria; 7 Institute of Organic Chemistry, Department of Natural Sciences and Sustainable Resources, 27270 BOKU University, Muthgasse 18, Vienna A-1190, Austria; 8 Institute of Molecular Modeling and Simulation, Department of Natural Sciences and Sustainable Resources, 27270 BOKU University, Muthgasse 18, Vienna A-1190, Austria; 9 Doctoral Programme BioToP - Biomolecular Technology of Proteins, 27270 BOKU University, Muthgasse 18, Vienna A-1190, Austria

## Abstract

A β-galactosidase (*Bbre*βgal-III) derived from the human isolate *Bifidobacterium breve* DSM 20213, which belongs to the glycoside hydrolase (GH) family 42, was successfully overproduced in *Escherichia coli*, purified to apparent homogeneity, and studied with respect to its biochemical and molecular properties as well as its transgalactosylation activity for the synthesis of galacto-oligosaccharides (GOS) in comparison to the previously reported two GH2 β-galactosidases, *Bbre*βgal-I and *Bbre*βgal-II, originating from this strain. *Bbre*βgal-III is a homotrimer with a molecular mass of approximately 240 kDa and shows distinct characteristics in terms of catalytic efficiency, pH dependence of activity and stability over a broad pH range, substrate specificities, and preference for the formation of specific GOS structures when compared with the other two GH2 β-galactosidases. These results suggest that multiple β-galactosidases in *B. breve* play divergent roles in substrate utilization under various conditions, especially in the degradation of various human milk oligosaccharide structures containing one or more β-linked galactose moieties as selective substrates for the growth and well-being of breast-fed infants. *Bbre*βgal-III displays transgalactosylation activity with a total GOS yield of 17% of total sugars with the preference to form 6′-galactosyllactose, which is also a predominant product during transgalactosylation of lactose. This finding paves the way for further work on this enzyme using engineering strategies to enhance transgalactosylation activity and hence the yield of the transgalactosylation products or for the biosynthesis of the GOS mixtures with specific structures, which could result in functionally enhanced prebiotic GOS.

## Graphical abstract

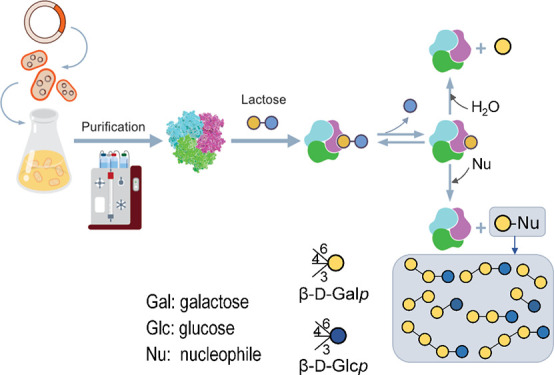

## Introduction

β-Galactosidases (EC 3.2.1.23), classified under different glycoside hydrolase families (GHs), catalyze the hydrolysis of various β-d-galactopyranosides (for example, lactose). One of the main technological applications of β-galactosidases in the dairy industry is the hydrolysis of lactose in milk and milk products as they can break down lactose into galactose and glucose. This not only clears up the lactose intolerance problem in consumers but also improves the sweetness, solubility, and digestibility of milk products.^1,2^ Furthermore, β-galactosidases also catalyze the transgalactosylation reaction of lactose, wherein a galactosyl moiety is transferred onto suitable acceptors, forming different galacto-oligosaccharides (GOS). GOS are characterized as nondigestible carbohydrates meeting the criteria of prebiotics. GOS have emerged as one of the primary prebiotics produced commercially and are often used in fortified infant formulas.^3–6^

In the process of lactose conversion catalyzed by β-galactosidases, two distinct steps occur (Scheme 1). First, a covalently linked galactosyl-enzyme intermediate is formed. Afterward, the galactosyl moiety from this complex is transferred to nucleophile acceptors. β-Galactosidases work in transgalactosylation mode when the galactosyl moiety is transferred to a sugar molecule and not to water.^2,7–9^ Transgalactosylation is described as a process involving both intramolecular and intermolecular reactions. The intramolecular transgalactosylation reaction involves the cleavage of the glycosidic β-d-(1→4) bond of lactose, which is immediately reformed at another position of the glucose molecule, for example as a β-d-(1→6) or β-d-(1→3) linkage, resulting in the formation of disaccharides. In an intermolecular transgalactosylation reaction, any sugar present in the reaction mixture can serve as a nucleophile; therefore, they can accept a galactosyl moiety from the galactosyl-enzyme complex to produce various di-, tri-, or even higher galacto-oligosaccharides. The production of GOS is not a result of an equilibrium reaction; instead, GOS products must be regarded as kinetic intermediates since they are also substrates for hydrolysis. Consequently, transgalactosylation reactions are kinetically controlled.^3^ The characteristics of produced GOS mixtures depend on their structural configuration, chemical composition, and degree of polymerization. It is widely recognized that β-galactosidases from different sources exhibit different specificity in building glycosidic linkages and hence produce different GOS mixtures.^10^

**Scheme 1:**
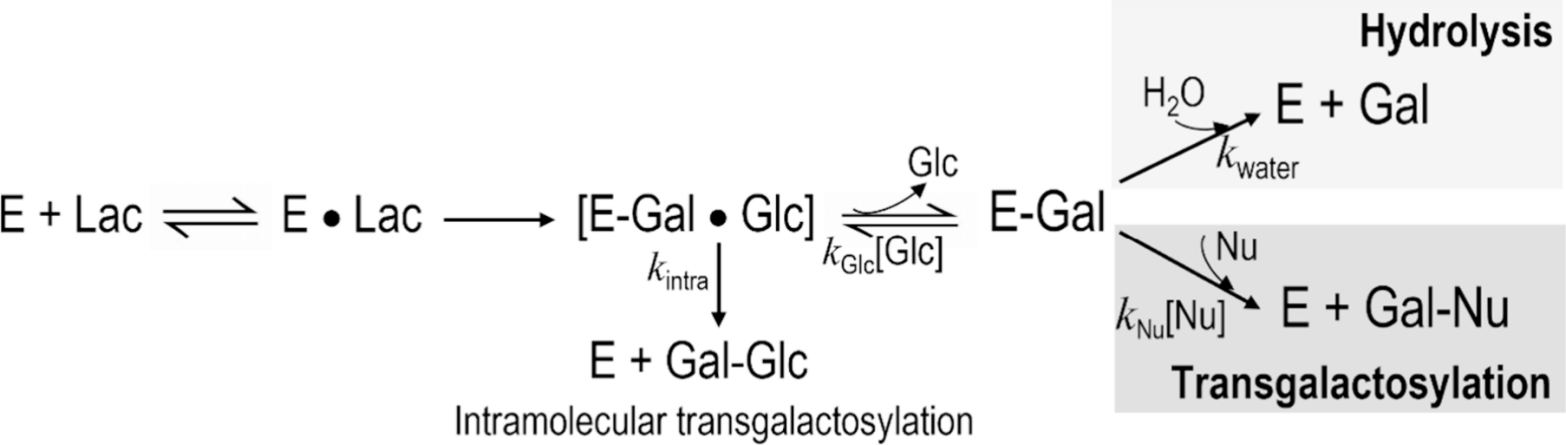
Hydrolysis and Transgalactosylation Reactions in the Process of Lactose Conversion Catalyzed by β-Galactosidases^a^

a E, enzyme; Lac, lactose; Gal, galactose; Glc, glucose; Nu, nucleophile; Gal-Nu, galactosyl adduct (GOS).

β-Galactosidases of the GH2 family generally receive more attention in terms of transgalactosylation activity and GOS formation in comparison to the GH42 β-galactosidases. The transgalactosylation activity of GH42 β-galactosidases has not yet been described in detail, but several members from this group of enzymes have been reported to be capable of synthesizing GOS using lactose as a substrate. In a study by Ambrogi et al., four GH42 β-galactosidases from *Bifidobacterium* spp. displayed transgalactosylation activity with varying GOS yields including *B. breve* UCC2003 (18.0%), *B. bifidum* LMG 13195 (13.7%), *B. longum* subsp. *longum* NCIMB 8809 (22.2%), and *B. longum* subsp. *infantis* ATCC 15697 (14.2%).^11^ Various hypotheses have been proposed to explain a sparse or even absent ability of GH42 β-galactosidases to synthesize GOS. Godoy et al. compared the structures between a GH42 β-galactosidase BbgII from *B. bifidum* and a GH2 β-galactosidase (LacZ) from *E. coli* and reported that the major catalytic residues (Glu161 and Glu320 in BbgII, Glu461 and Glu537 in LacZ) were identical in both enzymes; however, their catalytic properties are quite distinct, especially with respect to GOS synthesis.^12^ The authors argued that this distinction could be attributed to the molecular architecture of GH42 active sites, which favors binding of and inhibition by the α-d-anomer of galactose. In addition, it was demonstrated that in GH42 β-galactosidases, the galactose O1 exhibited a strong preference for forming interactions with Tyr289 rather than with the Glu161 side chain. In contrast, the galactose O1 in LacZ commonly interacts with the nucleophilic glutamic acid residue Glu537, typically in a β-d-anomeric form. Consequently, O1 becomes susceptible to nucleophilic attack from a galactoside leading to the formation of GOS.^12,13^

The genus *Bifidobacterium* holds significant importance in the human gastrointestinal tract (GIT), playing a vital role in maintaining gut health and overall well-being. Among *Bifidobacterium* species, *Bifidobacterium breve* is one of the most prevalent species found in the infant intestinal tract,^14^ and it has been widely used in pediatrics as a prominent probiotic in infants.^15^ The beneficial effect of gut bifidobacteria is attributed in part to their ability to metabolize human milk oligosaccharides (HMOs), which contain one or more β-linked galactose moieties, serving as selective substrates. It is known that GOS possess structural resemblance to oligosaccharides and diverse complex structures found in human breast milk. Among the glycosyl hydrolases from *Bifidobacterium* species involved in assimilation of different HMO structures, β-galactosidases are enzymes of great importance.^15–18^

Several studies have reported the presence of multiple β-galactosidases in *Bifidobacterium* species, for examples, in *B. infantis*, *B. adolescentis*, or *B. bifidum*.^19–21^ Several β-galactosidases derived from bifidobacteria have been studied for the biosynthesis of GOS.^19,21,22^ We have reported the overexpression and characterization of two GH2 β-galactosidases, *Bbre*βgal-I and *Bbre*βgal-II, from *B. breve* DSM 20213, which were found to show a high propensity to catalyze transgalactosylation reactions for the formation of GOS.^22^ In this present study, we describe the biochemical, molecular, and structural properties of the third β-galactosidase from *B. breve* DSM 20213 (*Bbre*βgal-III), which belongs to the GH42 family, with respect to the transgalactosylation activity and GOS synthesis in comparison with the other two GH2 β-galactosidases.

## Materials and Methods

### Chemicals and Vectors

All enzymes and chemicals were procured from Sigma (St. Louis, MO, USA), unless otherwise stated, and were of the highest quality available. Phusion high-fidelity DNA polymerase, restriction enzymes, T4 DNA ligase, and corresponding buffers were obtained from New England Biolabs (Frankfurt am Main, Germany), whereas DNA and protein standard ladders and staining dyes were sourced from Bio-Rad (Hercules, CA, USA). Isopropyl-β-d-thiogalactopyranoside (IPTG) was acquired from Roth (Karlsruhe, Germany). The Lactose Assay Kit-Sequential/High Sensitivity, l-Arabinose/d-Galactose Assay Kit, and Glucose (HK) Assay Kit used for the determination of d-lactose, d-galactose, and d-glucose, respectively, were purchased from Megazyme (Bray, Ireland). The plasmid pET21a­(+) was obtained from Novagen (Darmstadt, Germany).

### Bacterial Strains and Culture Conditions

*Bifidobacterium breve* DSM 20213 (ATCC 15700, NCTC 11815), sourced from the German Collection of Microorganisms and Cell Cultures (DSMZ; Braunschweig, Germany), was cultivated in De Man-Rogosa-Sharpe (MRS) medium (Carl Roth, Karlsruhe, Germany) at 37 °C under anaerobic conditions. *Escherichia coli* strains were grown at 37 °C with shaking at 200 rpm in Luria-Bertani (LB) medium supplemented with 100 μg mL^–1^ ampicillin. *E. coli* NEB5α (New England Biolabs, Ipswich, MA, USA) was used as the subcloning host for DNA fragments, while *E. coli* BL21 Star DE3 (Invitrogen, Carlsbad, CA, USA) was the expression host.

### Construction of the Expression Plasmid

Genomic DNA of *B. breve* DSM 20213, isolated using a Monarch Genomic DNA Purification Kit (New England Biolabs) according to the manufacturer’s instructions, was used as the template for the PCR reaction. Based on the sequence of the gene encoding the GH42 β-galactosidase (*Bbre*βgal-III) from *B. breve* DSM 20213 (BBBR_0453, GenBank genome sequence AP012324.1), the primer pairs were designed with the appropriate restriction sites, *Nde*I and *Not*I (underlined), introduced in the forward (AGATA **CATATG** GAACATCGCGAATTCAAGT) and reverse primers (AATAT **GCGGCCGC** CAGCTTTACCACCAGCACA), respectively. The primers were provided by Microsynth (Vienna, Austria). The gene was amplified by Phusion high-fidelity DNA polymerase. The amplification protocol consisted of the following steps: (1) denaturation at 98 °C for 3 min; (2) 35 cycles of denaturation at 98 °C for 10 s, annealing at 66 °C for 30 s, and extension at 72 °C for 75 s; (3) final extension at 72 °C for 2 min. A DNA Gel Extraction Kit (New England Biolabs) was used to purify the amplified product, which was then digested with the corresponding restriction enzymes. The digested fragment was ligated into pET21a­(+) resulting in the expression vector pET21a-*Bbre*βgal-III. *E. coli* NEB5α was used as the host for subcloning to obtain a sufficient amount of the plasmid. The sequence of the insert was verified through DNA sequencing provided by Microsynth. Subsequently, the expression vector pET21a-*Bbre*βgal-III was transformed into the expression host *E. coli* BL21 Star (DE3).

### Expression of the β-Galactosidase Gene

*E. coli* BL21 Star (DE3) harboring the plasmid pET21a-*Bbre*βgal-III was cultivated at 37 °C in 100 mL of LB broth containing 100 μg mL^–1^ ampicillin until an OD_600_ of 0.6 was reached. IPTG at various final concentrations (0.1, 0.5, and 1 mM) was added to the culture medium to evaluate the optimal IPTG concentration for inducing the expression of *Bbre*βgal-III. The cultures were further incubated at 25 °C and harvested at different time points after induction. The biomass was collected by centrifugation (4000*g*, 15 min, 4 °C), washed twice, and resuspended in 0.5 mL of 50 mM sodium phosphate buffer (NaPB) at pH 6.5. Cell disruption was performed on ice by sonication (Bandelin Sonopuls HD60, Berlin, Germany), followed by centrifugation (10,000*g*, 15 min, 4 °C) to obtain the crude recombinant enzyme. The activity of the recombinant β-galactosidase was determined by using the standard enzyme assay and protein concentration measurement.

### Production of Recombinant β-Galactosidase

*E. coli* BL21 Star (DE3) carrying the plasmid pET21a-*Bbre*βgal-III was cultivated at 37 °C in 300 mL of LB broth supplemented with 100 μg mL^–1^ ampicillin. When the OD_600_ reached 0.6, the cultures were induced by adding IPTG (0.1 mM), which was the optimal IPTG concentration obtained from the previous step, as described above, and the cultures were incubated further at 25 °C for 9 h. The biomass was harvested, washed twice, and resuspended with 50 mM NaPB (pH 6.5). Cells were then disrupted by using a French press (Aminco, Silver Spring, MD, USA). The cell debris was removed by centrifugation (25,000*g*, 30 min, 4 °C). The recombinant enzyme *Bbre*βgal-III was purified using a prepacked HiTrap IMAC HP 5 mL column (Cytiva, MA, USA). The crude extract was loaded on the column, which was pre-equilibrated with buffer A (20 mM NaPB, 20 mM imidazole, and 500 mM NaCl, pH 6.5). Subsequently, the His-tagged protein was eluted using buffer B (20 mM NaPB, 500 mM imidazole, and 500 mM NaCl, pH 6.5) with a 15 mL linear gradient from 0 to 100% at a rate of 1 mL min^–1^. Active fractions were then pooled, desalted, and concentrated through ultrafiltration using an Amicon Ultra Centrifugal Filter Unit with a 30 kDa cutoff membrane (Millipore, MA, USA).

### Determination of Molecular Mass

#### Gel Electrophoresis Analysis

The purity and molecular mass of recombinant *Bbre*βgal-III were determined by SDS-PAGE. The enzyme with a protein concentration of 1.5 mg mL^–1^ was denatured using Laemmli buffer at 90 °C for 5 min, followed by electrophoresis using Mini-PROTEAN TGX Stain-Free Protein Gel (Bio-Rad) at 100 V for 45 min. The resulting gel was visualized using a Molecular Imager Gel Doc XR system (Bio-Rad).

#### Size-Exclusion Chromatography (SEC)

The molecular weight and oligomeric state of *Bbre*βgal-III were determined by an OMNISEC multidetector GPC/SEC system (Malvern Panalytical, Vienna, Austria). The sample (in NaPB, pH 6.5) was centrifuged at 16,000*g* for 10 min and then filtered through a Durapore PVDF centrifugal filter (0.1 μm). Subsequently, 22 μL of the sample was injected at a concentration of 13.4 mg mL^–1^ with a flow rate of 0.5 mL min^–1^ at 20 °C. The solvent was Dulbecco’s phosphate-buffered saline (DPBS) without Ca^2+^ and Mg^2+^ (PAN-Biotech, Aidenbach, Germany). As the sample eluted, it was analyzed by three detectors in series, namely, a refractive index (RI) detector, a diode-array-based UV/Vis spectrometer recording the absorbance of a sample at any wavelength ranging from 190 to 900 nm, and a light scattering detector that combines 90° right-angle light scattering (RALS) with 7° low-angle light scattering (LALS). Data analysis was performed by OMNISEC software v11.37. The average dn/dc value of 0.185 mL/g was used in the calculation.^23,24^

### β-Galactosidase Assays

The enzyme activity was measured using two substrates, *o*-nitrophenyl-β-d-galactopyranoside (*o*NPG) or lactose, as previously described.^25^ In the assay with the chromogenic substrate *o*NPG, 20 μL of enzyme solution was added to 480 μL of 22 mM *o*NPG (in 50 mM NaPB, pH 6.5). The mixture was incubated at 30 °C for 10 min. After incubation, the reaction was stopped by adding 750 μL of 0.4 M Na_2_CO_3_. The release of *o*-nitrophenol (*o*NP) was determined by measuring the absorbance at 420 nm. One unit of *o*NPG activity was defined as the amount of β-galactosidase liberating 1 μmol of *o*NP per minute under the described conditions.

In the assay with lactose as the substrate, the reaction mixture consisted of 20 μL of the enzyme solution and 480 μL of 600 mM lactose solution (in 50 mM NaPB, pH 6.5). The reaction mixture was incubated at 30 °C for 10 min and then stopped by heating at 99 °C for 5 min. After cooling to room temperature, the release of d-glucose in the reaction was measured using a Glucose (HK) Assay Kit (Megazyme) following the manufacturer’s instructions. One unit of lactase activity was determined as the amount of β-galactosidase releasing 1 μmol of d-glucose per minute under the specified conditions.

### Determination of Protein Concentration

Protein concentrations were determined according to the method of Bradford^26^ using bovine serum albumin as the standard.

### Biochemical Characterization of Recombinant β-Galactosidase

All steady-state kinetic measurements were conducted in 50 mM NaPB (pH 6.5) at 30 °C using *o*NPG or lactose as the substrates with the concentrations of *o*NPG ranging from 0.01 to 22 mM and lactose concentrations ranging from 10 to 600 mM. The kinetic constants and parameters were calculated based on nonlinear regression, in which the obtained data were fit to the Henri-Michaelis-Menten equation using the data analysis software SigmaPlot (SPSS, IL, USA). The substrate inhibition constant (*k*_i,S_) was calculated by using the built-in model in SigmaPlot for substrate inhibition: *v* = *v*_max_/(1 + *K*_m_/[S] + [S]/*k*_i,S_).

The effects of pH and temperature on *Bbre*βgal-III activity were evaluated under specified conditions by performing standard assays with 22 mM *o*NPG and 600 mM lactose as the substrates. In the pH dependency measurements, the substrate solutions were prepared in Britton-Robinson buffer (20 mM acetic acid, 20 mM phosphoric acid, and 20 mM boric acid) titrated with 1 M NaOH to obtain the desired pH values ranging between pH 3 and pH 9. In the temperature dependency measurements, the substrates were prepared in 50 mM NaPB (pH 6.5) and the activity assays were performed at temperatures ranging from 20 to 80 °C.

The recombinant enzyme *Bbre*βgal-III was incubated in Britton-Robinson buffer at different pH values (pH 4–9) at 37 °C for pH stability assessment. Thermostability was determined by incubating the enzyme in 50 mM NaPB (pH 6.5) at various temperatures between 4 and 80 °C. In addition, the effect of magnesium ions (Mg^2+^) on thermostability of *Bbre*βgal-III was also evaluated by incubating the enzyme in the presence of 1 and 10 mM MgCl_2_ at 45, 50, and 55 °C. At certain time intervals, samples were withdrawn to measure the remaining enzyme activity, and the half-life (τ_1/2_) value was determined when the retaining enzyme activity was 50% of its initial activity.

Differential scanning calorimetry (DSC) measurements were performed by using a MicroCal PEAQ-DSC Automated system (Malvern Panalytical). The purified enzyme sample was prepared in 10 mM Tris buffer with a protein concentration of 7.7 mg mL^–1^ (∼98 μM). The reference cell (325 μL) was initially filled with 10 mM Tris buffer, and the sample cell (325 μL) was filled with the enzyme sample. These cells were then heated in the range of 20–80 °C at a constant scan rate of 60 °C h^–1^. A thermodynamic model was fit to the data to determine the change in the heat capacity (Δ*C_p_*) associated with the transition.

### Substrate Preference

Different galactosides (β-d-Gal*p*-(1→4)-d-Lac, β-d-Gal*p*-(1→6)-d-Lac, β-d-Gal*p*-(1→3)-d-Lac, β-d-Gal*p*-(1→4)-d-Gal, β-d-Gal*p*-(1→6)-d-Gal, β-d-Gal*p*-(1→3)-d-Gal, β-d-Gal*p*-(1→6)-d-Glc, β-d-Gal*p*-(1→3)-d-Glc, and β-d-Gal*p*-(1→4)-d-Glc) were used to determine the substrate preference of recombinant *Bbre*βgal-III. Approximately 3 mM of each substrate was incubated with an appropriate amount of *Bbre*βgal-III at 30 °C in 50 mM NaPB, pH 6.5, for 60 min. The reactions were stopped by heating at 95 °C for 5 min, and the resulting sugars were quantitated by HPAEC-PAD (see below). The rates of the conversion of each galactoside by *Bbre*βgal-III are expressed relatively to the conversion of lactose (as 100%) after 60 min under the same conditions.

### GOS Synthesis

The transgalactosylation reaction of lactose by *Bbre*βgal-III was conducted at 37 °C using a 215 g L^–1^ lactose solution in 50 mM sodium phosphate buffer (pH 6.5). Samples were collected at certain intervals for the analysis of the carbohydrate contents of the reaction mixtures. Lactose, galactose, and glucose concentrations were determined using the Lactose Assay Kit-Sequential/High Sensitivity, l-Arabinose/d-Galactose Assay Kit, and Glucose (HK) Assay Kit (Megazyme), respectively, according to the manufacturer’s guidelines.

### Analysis of the GOS Mixtures

#### High-Performance Anion-Exchange Chromatography with Pulsed Amperometric Detection (HPAEC-PAD)

HPAEC-PAD analysis to determine the carbohydrate content in the reaction mixtures was carried out using a Dionex DX-500 system as previously described in detail.^3^

#### Ultrahigh-Performance Liquid Chromatography–Mass Spectrometry (UPLC-MS)

The spectrum of the products formed in terms of the degree of polymerization (DP) was determined by ultrahigh-performance liquid chromatography-mass spectrometry (UPLC-MS) including a Vanquish Horizon UHPLC System and an Orbitrap IQ-X Tribid mass spectrometer (Thermo Fisher Scientific, Bremen, Germany). Thermo Xcalibur version 4.2 (Thermo Fisher Scientific) was used for instrument control, data handling, and processing. A linear gradient from 1 to 25% B (A = 0.1% formic acid; B = 0.1% formic acid in 95% acetonitrile) at a flow rate of 300 μL min^–1^ was applied (9 min gradient time) followed by a 1 min linear gradient from 25 to 75% B using a Supelco Supel Carbon LC Guard Cartridge (20 × 2.1 mm, 2.7 μm). Detection was performed with an Orbitrap instrument (LC-OTMS^n^, Orbitrap IQ-X Tribid, Thermo Fisher Scientific) equipped with the standard ESI source (HESI probe) in positive ionization mode, MS/MS mode (range: 100-1200 *m*/*z*). Instrument calibration was performed by using an integrated Auto-Ready ion source and an Internal Thermo Scientific Pierce FlexMix Calibration solution. A blank sample was prepared using deionized water. Detailed information about UPLC-Orbitrap MS for analysis of oligosaccharides is listed in Table S1 (Supporting Information).

#### High-Performance Size-Exclusion Chromatography with UV-Based Detection (HPSEC-UV)

Size distribution analysis of GOS samples (2 mL injections of ∼37 mg mL^–1^ GOS in NaPB, pH 6.5) was performed on a Bio-Gel P2 column (Column XK 16/100, Cytiva, packed with Bio-Gel P2 Gel, Bio-Rad, CA, USA). The column was eluted with distilled water at a flow rate of 0.15 mL min^–1^. The elution was monitored by UV detection at 190 nm to detect the transgalactosylation products. Fractions containing different DP of GOS were then pooled and lyophilized for NMR spectroscopy analysis.

#### NMR Spectroscopy

NMR spectra were recorded with a Bruker Avance III 600 instrument (600.22 MHz for ^1^H, 150.93 MHz for ^13^C) using the standard Bruker NMR software. ^1^H spectra were recorded in D_2_O at 300 K. Assignments were based on COSY, HSQC, and HMBC data. ^1^H spectra were referenced externally to acetone in D_2_O (2.225 ppm).

### Structure Prediction

The structure of the multimeric enzyme *Bbre*βgal-III was predicted by AlphaFold database version 2.2.4.^27^ To identify the critical residues in the active site, the predicted structure of *Bbre*βgal-III was aligned with the crystal structure of *Bifidobacterium bifidum* S17 β-galactosidase (PDB 4UCF) using the PyMOL Molecular Graphics System (version 2.4, Schrödinger, LLC).

### Statistical Analysis

All measurements were carried out in triplicate, and the data were expressed as the mean ± SD (standard deviation). The standard deviations were always less than 5%.

## Results

### Production of the Recombinant GH42 β-Galactosidase from *B. breve* (*Bbre*βgal-III) in *E. coli*

*E. coli* BL21 Star (DE3) harboring the expression vector pET21a-*Bbre*βgal-III was cultivated in LB medium, and the expression of *Bbre*βgal-III was induced using IPTG (Figure S1A). The highest yield, 497 kU L^–1^ medium with a specific activity of 947 U mg^–1^, was obtained after 9 h of induction when 0.1 mM IPTG was used. The enzyme was purified using a HiTrap IMAC HP 5 mL column (Cytiva), after which the specific activity of purified *Bbre*βgal-III increased to 1500 U mg^–1^. The purification step yielded an apparently homogeneous preparation of *Bbre*βgal-III with a molecular mass of approximately 77 kDa as judged by SDS-PAGE in comparison with reference proteins (Figure S1B). Furthermore, size-exclusion chromatography with an integrated triple-detector system (refractive index, light scattering, and UV/Vis PDA) revealed that the molecular mass of *Bbre*βgal-III is approximately 240 kDa (Figure S2). Therefore, it could be concluded that *Bbre*βgal-III is likely a homotrimer with three identical subunits, 77 kDa each.

### Biochemical Properties of *Bbre*βgal-III

The steady-state kinetic constants of recombinant *Bbre*βgal-III determined for both *o*NPG and lactose hydrolysis are summarized in Table 1. The *k*_cat_ values were calculated based on *v*_max_ values experimentally assessed by nonlinear regression and using a molecular mass of 77.3 kDa for the catalytically active subunit. The catalytic efficiency (*k*_cat_/*K*_m_) with *o*NPG is ∼700-fold higher than that of lactose, indicating that *o*NPG is the preferred substrate for *Bbre*βgal-III. In addition, substrate inhibition was observed in the hydrolysis of *o*NPG (*k*_i,S_ ≈ 13.8 mM), whereas the enzyme was not inhibited by its natural substrate lactose at concentrations up to 600 mM.

**Table 1:** Kinetic Parameters of Recombinant β-Galactosidase *Bbre*βgal-III from *B. breve* for the Hydrolysis of *o*-Nitrophenyl-β-d-galactopyranoside (*o*NPG) and Lactose^a^

substrate	*v*_max_ (μmol min^–1^ mg^–1^)	*K*_m_ (mM)	*k*_cat_ (s^–1^)	*k*_cat_/*K*_m_ (mM^–1^ s^–1^)	*k*_i,S_ (mM)
*o*NPG	3650 ± 200	1.57 ± 0.16	4690 ± 260	2990 ± 140	13.84 ± 1.57
lactose	84 ± 2	25.4 ± 1.4	108 ± 3	4.3 ± 0.1	

a Steady-state kinetic measurements were conducted in 50 mM NaPB (pH 6.5) at 30 °C using *o*NPG or lactose as the substrates.

The optimum pH of *Bbre*βgal-III activity was 5.0 for *o*NPG hydrolysis and 5.0–6.5 for lactose hydrolysis (Figure 1A). *Bbre*βgal-III exhibited more than 75% of its maximum activity within the pH range of 4.5–6.5 for *o*NPG hydrolysis and 3.5–7.0 for lactose hydrolysis. The highest enzyme activity was observed at 55 °C for *o*NPG hydrolysis, which was higher than the optimum temperature at 40 °C for lactose hydrolysis under the standard assay conditions (Figure 1B). The enzyme activities at 45 and 50 °C for lactose hydrolysis were 92 and 86%, respectively, of its maximum activity at 40 °C.

**Figure 1:**
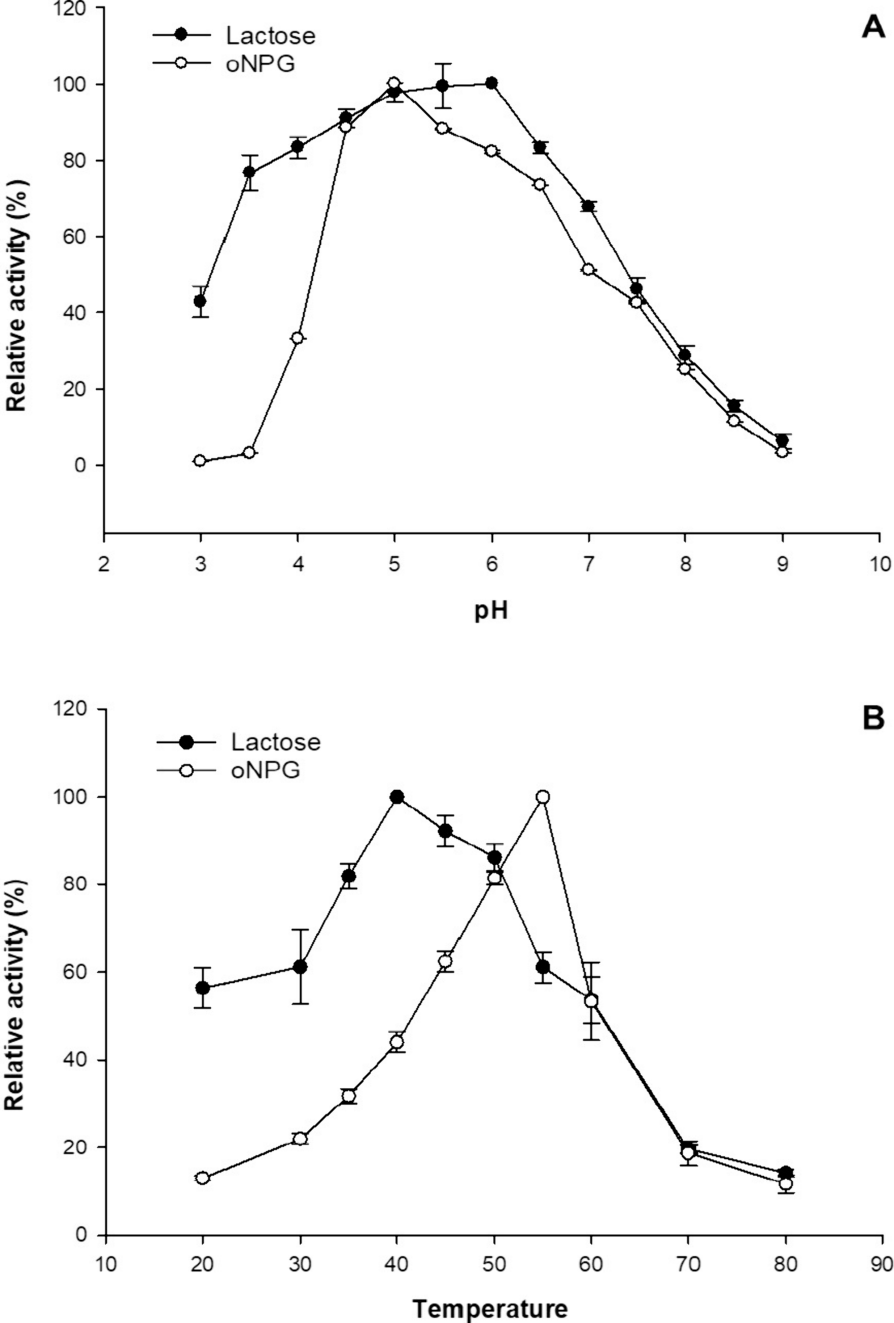
pH (A) and temperature optimum (B) of *Bbre*βgal-III activity using *o*-nitrophenyl-β-d-galactopyranoside (*o*NPG) and lactose as the substrates. Relative activities were calculated by comparing with the maximum activity measured under optimal conditions (100%). All data points are the mean ± SD (standard deviation) of at least two independent replicates, and the standard deviations were always less than 5%.

*Bbre*βgal-III demonstrated excellent stability over a broad pH range, and it is most stable at pH 5.0 with a half-life time of activity (τ_1/2_) of 67 days at 37 °C (Table S2). The enzyme was inactivated rapidly under strongly acidic conditions with pH values below 3.0. The enzyme was stable at temperatures ranging from 20 to 50 °C, retaining more than 80% of its activity at 37 and 50 °C after 27 and 7 days, respectively (Figure S3A,B). The long-term storage of the enzyme was achieved at 4 °C (τ_1/2_ > 2.5 years)_._ Temperature exceeding 60 °C led to rapid inactivation of enzymatic activity. The presence of Mg^2+^ considerably improved the thermostability of *Bbre*βgal-III (Figure S3B). At 55 °C, the τ_1/2_ of *Bbre*βgal-III was 5 h; however, when the enzyme was incubated in 50 mM NaBP (pH 6.5) in the presence of 1 and 10 mM MgCl_2_, the half-life time of activities τ_1/2_ increased to ∼10 and ∼20 h, respectively (Figure S3B). A further study by differential scanning calorimetry (DSC) analysis of purified *Bbre*βgal-III showed a single endothermic peak, which fitted very well based on a two-state transition model, and the observed melting temperature (*T*_m_) of 58 °C (data not shown) was in agreement with the optimum temperature of the enzyme as shown in Figure 1B.

### Substrate Preference

The activities of *Bbre*βgal-III with various galactosides were determined and compared with those of two GH2 β-galactosidases of the LacZ-type, *Bbre*βgal-I and *Bbre*βgal-II, from *B. breve* DSM 20213 ^22^ (Figure 2). Activities of each enzyme with the individual substrates are expressed as relative conversion (in percentage, %) of the conversion of lactose after 60 min. *Bbre*βgal-I and *Bbre*βgal-II exhibited high activities toward lactose and certain substrates such as β-d-Gal*p*-(1→6)-d-Glc (allolactose) and β1→3-linked substrates, which were converted at comparable rates, whereas β-d-Gal*p*-(1→6)-d-Lac and the β1→4-linked substrates (except lactose) including β-d-Gal*p*-(1→4)-d-Lac and β-d-Gal*p*-(1→4)-d-Gal were converted at significantly lower rates. Interestingly, a different pattern was observed in the case of *Bbre*βgal-III. Instead of lactose, *Bbre*βgal-III displayed significantly higher preferences toward some other tested oligosaccharides. The β1→3-linked substrates including β-d-Gal*p*-(1→3)-d-Lac, β-d-Gal*p*-(1→3)-d-Gal, and β-d-Gal*p*-(1→3)-d-Glc were converted five times faster compared to the conversion of lactose. Other substrates, including β-d-Gal*p*-(1→6)-d-Lac, β-d-Gal*p*-(1→6)-d-Gal, and β-d-Gal*p*-(1→4)-d-Lac, were also converted at significantly higher rates in comparison with lactose, whereas allolactose and lactose were converted at comparable rates. In addition, the disaccharide β-d-Gal*p*-(1→4)-d-Gal was converted at slow rates by all three enzymes.

**Figure 2:**
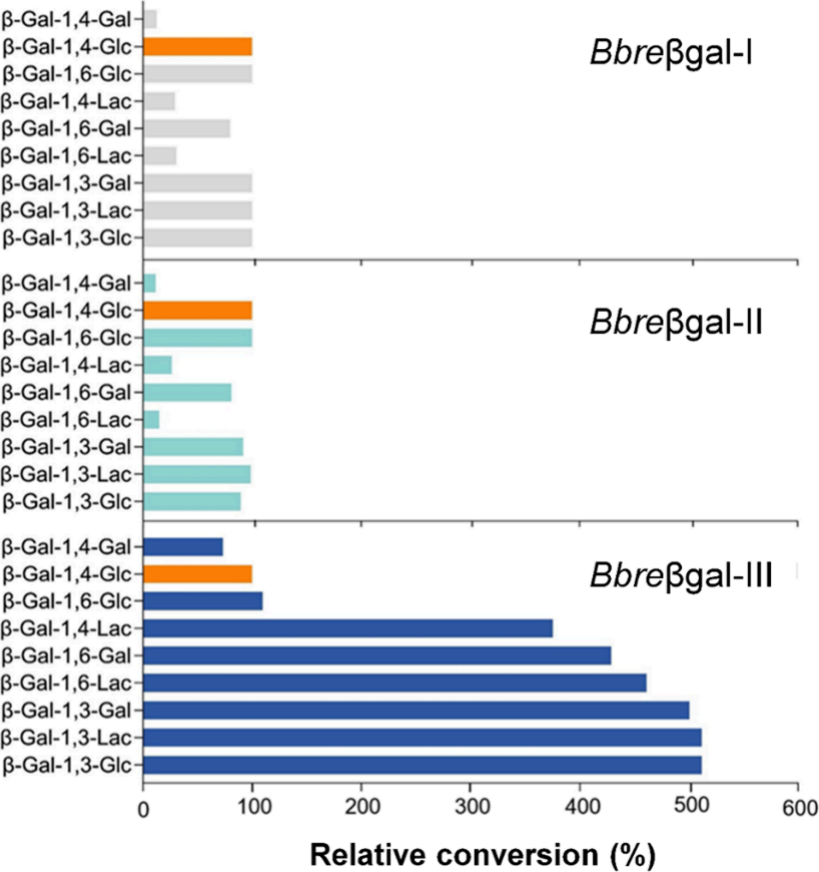
Relative activities of the β-galactosidases from *B. breve* DSM 20213 for individual galactosides. The rates of the conversion of each galactoside are expressed relative to the conversion of lactose, β-d-Gal*p*-(1→4)-d-Glc, (as 100%; in orange) after 60 min under the same conditions (at 30 °C in 50 mM sodium phosphate buffer, pH 6.5).

### GOS Synthesis and Identification of Individual Components

Carbohydrate formation of a typical discontinuous conversion at 37 °C, using an initial lactose concentration of 205 g L^–1^ (or 600 mM) in 50 mM sodium phosphate buffer (pH 6.5), is displayed in Figure 3A. Under these conditions, a maximum GOS concentration of 37 g L^–1^ was obtained, accounting for 17% of total sugars in the reaction mixture, when lactose conversion reached approximately 70%.

**Figure 3:**
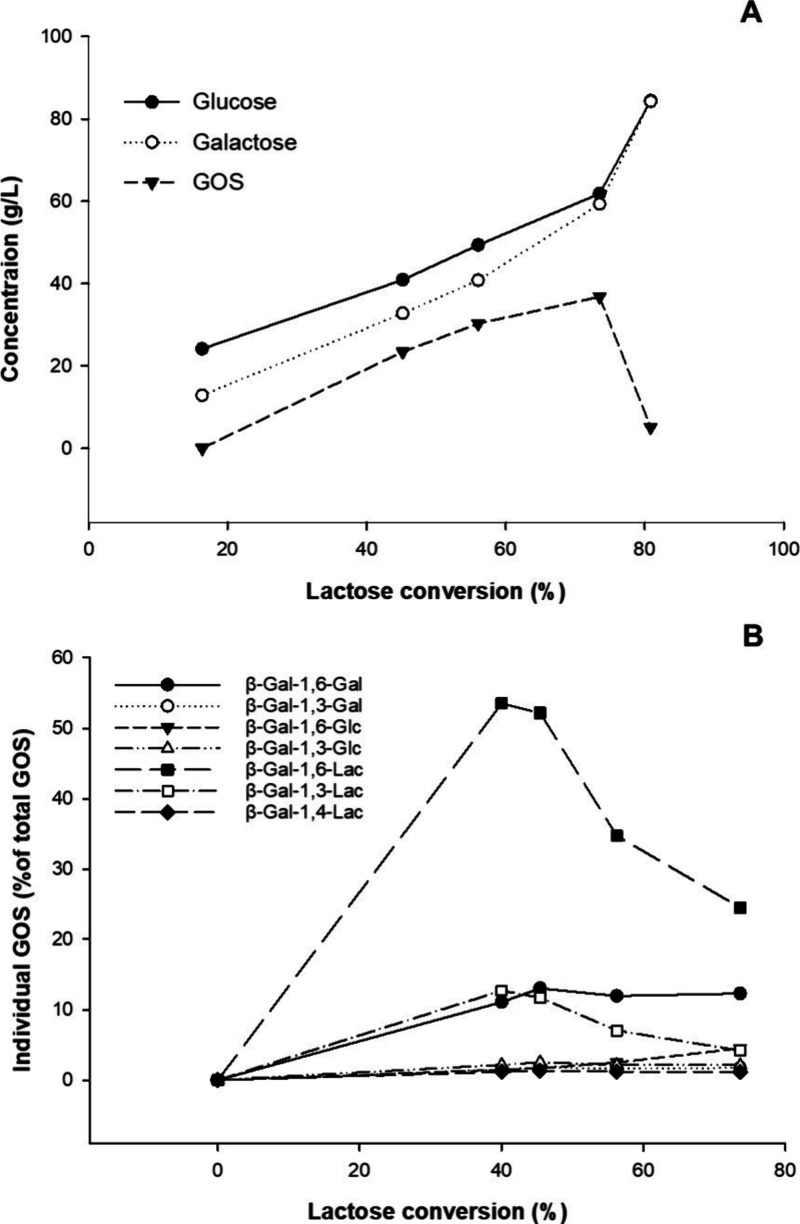
Carbohydrate formation (A) and formation as well as degradation of individual GOS (B) during lactose conversion catalyzed by *Bbre*βgal-III at 37 °C with an initial lactose concentration of 215 g L^–1^ in 50 mM sodium phosphate buffer (pH 6.5).

The GOS produced are not the products of an equilibrium reaction but must be regarded as kinetic intermediates as they are also substrates for hydrolysis. Although the highest GOS yield was obtained at ∼70% lactose conversion, the degradation (or hydrolysis) of some GOS components was already observed after 40% of lactose conversion catalyzed by *Bbre*βgal-III (Figure 3B). The partitioning of the galactosylated enzyme (which is formed as an intermediate in the enzymatic reaction; E-Gal in Scheme 1) between reaction with water and hence hydrolysis and reaction with the sugar acceptors can be studied under initial velocity conditions.^28^ When the galactosyl moiety is transferred to water (Scheme 1), hydrolysis occurs; hence, the hydrolytic activity can be calculated using the equation: Hydrolysis (%) = [Gal]/[Glc] × 100. During transgalactosylation, the galactosyl moiety is transferred to galactosyl acceptors to form GOS (Scheme 1) and the transgalactosylation activity can be calculated as: Transgalactosylation (%) = ([Glc] – [Gal])/[Glc] × 100, where [Glc] and [Gal] are glucose and galactose concentrations (in g L^–1^), respectively. Therefore, it could be determined that at ∼40% lactose conversion, *Bbre*βgal-III displayed ∼78% hydrolytic activity and ∼22% transgalactosylation activity, hence a preference for hydrolysis over transgalactosylation.

Individual GOS components were separated by HPAEC-PAD, allowing the identification of the main transgalactosylation products. At approximately 40% lactose conversion, the predominant product was identified as β-d-Gal*p*-(1→6)-d-Lac, accounting for approximately 56% of the total GOS formed, followed by β-d-Gal*p*-(1→3)-d-Lac and β-d-Gal*p*-(1→6)-d-Gal, contributing approximately 13 and 12% of the total GOS produced, respectively (Figure 3B). Other identified products, β-d-Gal*p*-(1→3)-d-Gal, β-d-Gal*p*-(1→3)-d-Glc, β-d-Gal*p*-(1→4)-d-Lac, and β-d-Gal*p*-(1→6)-d-Glc (allolactose), make up approximately 7% of the total GOS. The remaining 12% of the total GOS was contributed by other products, which were unidentified by HPAEC-PAD.

UPLC-MS analysis confirmed that the GOS mixture produced by *Bbre*βgal-III also contained tetrasaccharides (DP4) (Figure 4A). The separated fractions after size-exclusion chromatography (HPSEC-UV) representing the tri- and tetrasaccharide fractions were further analyzed by NMR (Figure 4B). Reporter signals were compared with extensive literature data^29,30^ and cross-checked via ^1^H-^13^C HSQC, COSY, and HMBC experiments for plausibility.

**Figure 4:**
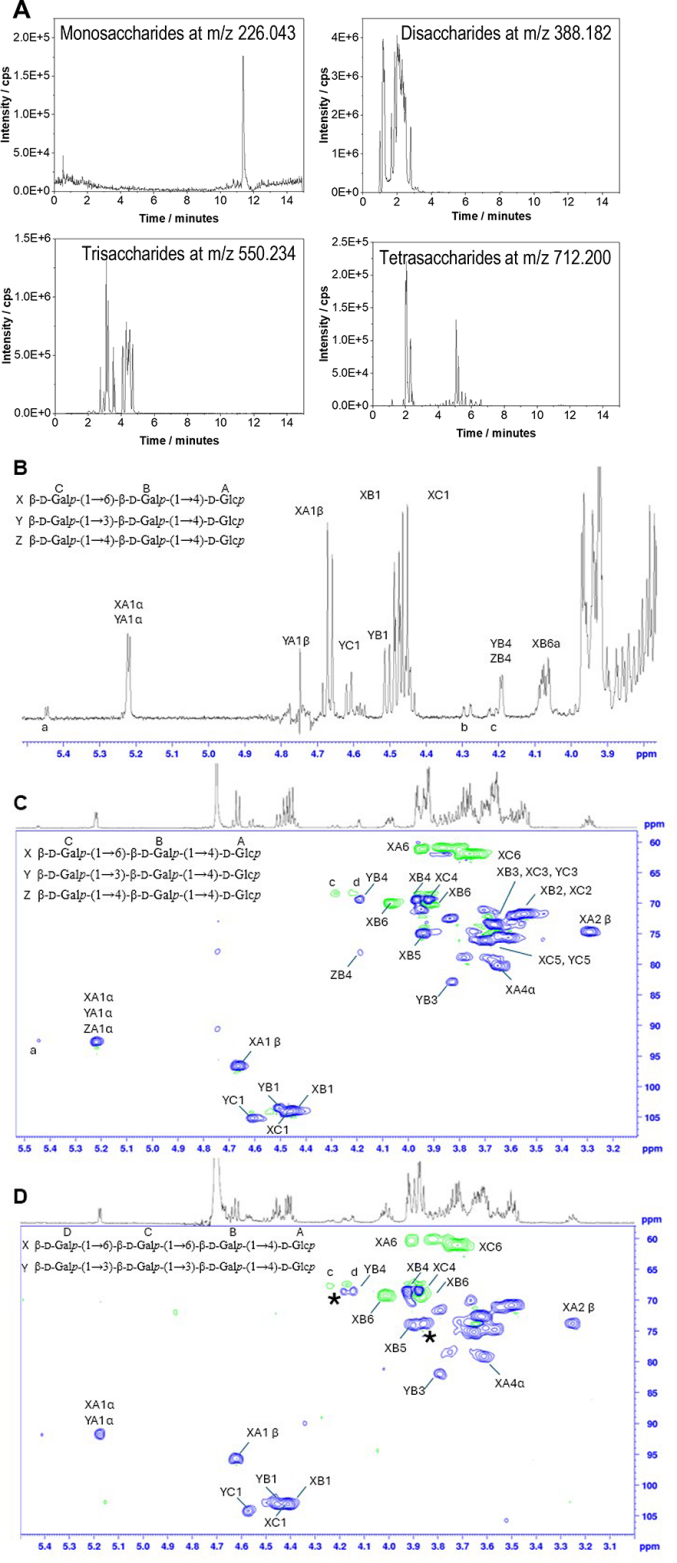
Analysis of GOS mixtures formed by *Bbre*βgal-III using UPLC-MS (A) and NMR spectroscopy (B, C, and D). (A) Extracted ion chromatogram of mono-, di-, tri-, and tetrasaccharides. Identification of the degrees of polymerization (DPs) in the samples was based on the accurate mass-to-charge ratio (*m*/*z*) of protonated molecules (in (+) ESI mode or positive electrospray ionization mode) and adducts. (B) ^1^H NMR analysis of the reporter region from the trisaccharide fraction. (C) ^1^H-^13^C HSQC analysis of the trisaccharide fraction and (D) ^1^H-^13^C HSQC analysis of the tetrasaccharide fraction. Differences to the trisaccharide fraction are indicated with the (*).

For the trisaccharide fraction, the two main components were found to be β-d-Gal*p*-(1→6)-β-d-Gal*p*-(1→4)-d-Glc*p* and β-d-Gal*p*-(1→3)-β-d-Gal*p*-(1→4)-d-Glc*p*. The indicative Gal H-4 signal of -3)-β-d-Gal*p*-(1- was overlaying with the H-4 of β-d-Gal*p*-(1→4)-β-d-Gal*p*-(1→4)-d-Glc*p* in the ^1^H dimension, but the presence of the 1-4 linkage could be shown in the ^13^C dimension (78.05 ppm vs 69.37 ppm) (Figure 4C). Furthermore, two interesting partial structures, of which the full trisaccharide structure could not be fully assigned, were identified. The signal at 5.45 ppm in the ^1^H dimension is indicative of an H-1 of a 2-substituted α-d-Glc unit^29,30^ (Figure 4B), while the signals at 4.28 and 4.21 ppm are indicative signals of a 6-substituted α/β-d-Glc*p* unit^29,30^ (Figure 4B).

While the ^1^H spectrum of the tetrasaccharide fraction showed to be more challenging than the trisaccharide fraction (Figure 4D), the HSQC spectra, in comparison with the trisaccharide fractions’ HSQC spectra, gave valuable insight into the likely composition of this fraction. Analyzing new cross-peaks and comparing relative intensities between the tri- and tetrasaccharide fractions, it appeared that the majority of the tetrasaccharide fraction consisted of two main molecules with relative intensities comparable to each other, as observed for the trisaccharide fraction. When overlaying the HSQC spectra, the main peaks of both fractions were found to be almost identical with notable differences only in the appearance of two additional signals, namely, 4.23/68.6 and 3.90/73.3. Whereas the signal at ^1^H 4.23 ppm had HSQC cross-peak intensities comparable to the minor compound, the signal at 3.90 ppm had intensities relative to the major compound. Since the minor compound in the trisaccharide fraction was found to be β-d-Gal*p*-(1→3)-β-d-Gal*p*-(1→4)-d-Glc*p* and the major compound was β-d-Gal*p*-(1→6)-β-d-Gal*p*-(1→4)-d-Glc*p* in HPAEC-PAD analysis, we therefore assumed that the two new signals would be based on the chemical shift and intensity attributable to an additional H-4 of a β-d-Gal*p*-(1→3) unit and H-5 of a β-d-Gal*p*-(1→6)-unit, which would lead to two components being β-d-Gal*p*-(1→6)-β-d-Gal*p*-(1→6)-β-d-Galp-(1→4)-d-Glc*p* (6′,6′-digalactosyllactose) as the major tetrasaccharide and β-d-Gal*p*-(1→3)-β-d-Gal*p*-(1→3)-β-d-Gal*p*-(1→4)-d-Glc*p* (3′,3′-digalactosyllactose) as the minor one, although thorough analysis of isolated compounds would be necessary to draw a final conclusion. Additionally, the same signals at 4.28 and 4.21 ppm, which are indicative signals of a 6-substituted α/β-d-Glc*p* unit and seen in the trisaccharide fraction, were also found in the tetrasaccharide fraction. We therefore assume that the 6-substituted trisaccharide was further converted to a tetrasaccharide.

### Structure Prediction of *Bbre*βgal-III

Based on protein structure modeling by the AlphaFold database (version 2.2.4), *Bbre*βgal-III is predicted to be a homotrimer as shown in Figure 5A, left. This finding corresponds to the crystal structures of other GH42 β-galactosidases in the PDB database such as *Bi*Bga42A from *B. longum* subsp. *infantis* (PDB 8IBT), BbgII from *B. bifidum* S17 (PDB 4UCF), Gan42B from *Geobacillus stearothermophilus* (PDB 5DFA), and BIGal42A from *B. animalis* subsp. *lactis* Bl-04 (PDB 4UNI). The GH42 β-galactosidases from *B. bifidum* S17 (BbgII, PDB 4UCF) and *B. longum* subsp. *infantis* (*Bi*Bga42A, PDB 8IBT) were selected as the templates. An overlay of the two structures, *Bbre*βgal-III and BbgII from *B. bifidum* S17 (PDB 4UCF), was performed to identify the active site of *Bbre*βgal-III, showing the residues located close to the galactose molecule. The active site of *Bbre*βgal-III has a deep narrow entrance and is located not far off the surface between each subunit (Figure 5A, middle). The crystal structure of the template (*B. bifidum* S17, PDB 4UCF) revealed that Glu161 and Glu320 are crucial residues for catalysis.^12^ The predicted model of *Bbre*βgal-III showed that Glu160 and Glu318 are the acid/base and the nucleophile residues, respectively (Figure 5A, right). The overlaid structure of the active site of *Bbre*βgal-III with the active sites of the GH42 *Bi*Bga42A from *B. longum* subsp. *infantis* ATCC 15697 (PDB 8IBT) and GH42 BbgII from *B. bifidum* S17 (PDB 4UCF) shows the conservation of the two catalytic glutamic acid residues, Glu160 and Glu318.^12,31^ The distance between C1 of the galactose moiety and the nucleophile (Glu318) is 3.3 Å, while the anomeric hydroxyl group is 4 Å from the acid/base residue (Glu160). These proximities are consistent with the catalytic geometry of a double-displacement mechanism in anomer-retaining glycoside hydrolases, where the nucleophile attacks the anomeric carbon to form a covalent glycosyl-enzyme intermediate, and the acid/base residue facilitates glycosidic bond cleavage and formation, as described by Koshland.^32^ The predicted model of *Bbre*βgal-III was also overlaid with the crystal structure of *Bi*Bga42A from the *B. longum* subspecies *infantis* ATCC 15697 (PDB 8IBT), which contained the tetrasaccharide β-d-Gal-(1→3)-β-d-GlcNAc-(1→3)-β-d-Gal-(1→4)-d-Glc (lacto-*N*-tetraose, LNT) as a ligand (Figure 5B, left). The crystal structure of the template (*Bi*Bga42A, PDB 8IBT) complexed with LNT revealed that glucose and galactose (in the lactose moiety, β-d-Gal-(1→4)-d-Glc) and *N*-acetylglucosamine (in the lacto-*N*-biose moiety, LNB, β-d-Gal-(1→3)-β-d-GlcNAc) of LNT occupy the +3, +2, and +1 subsites, respectively. The galactose in the LNB moiety of LNT is in the −1 subsite (Figure 5B, right).^31^

**Figure 5:**
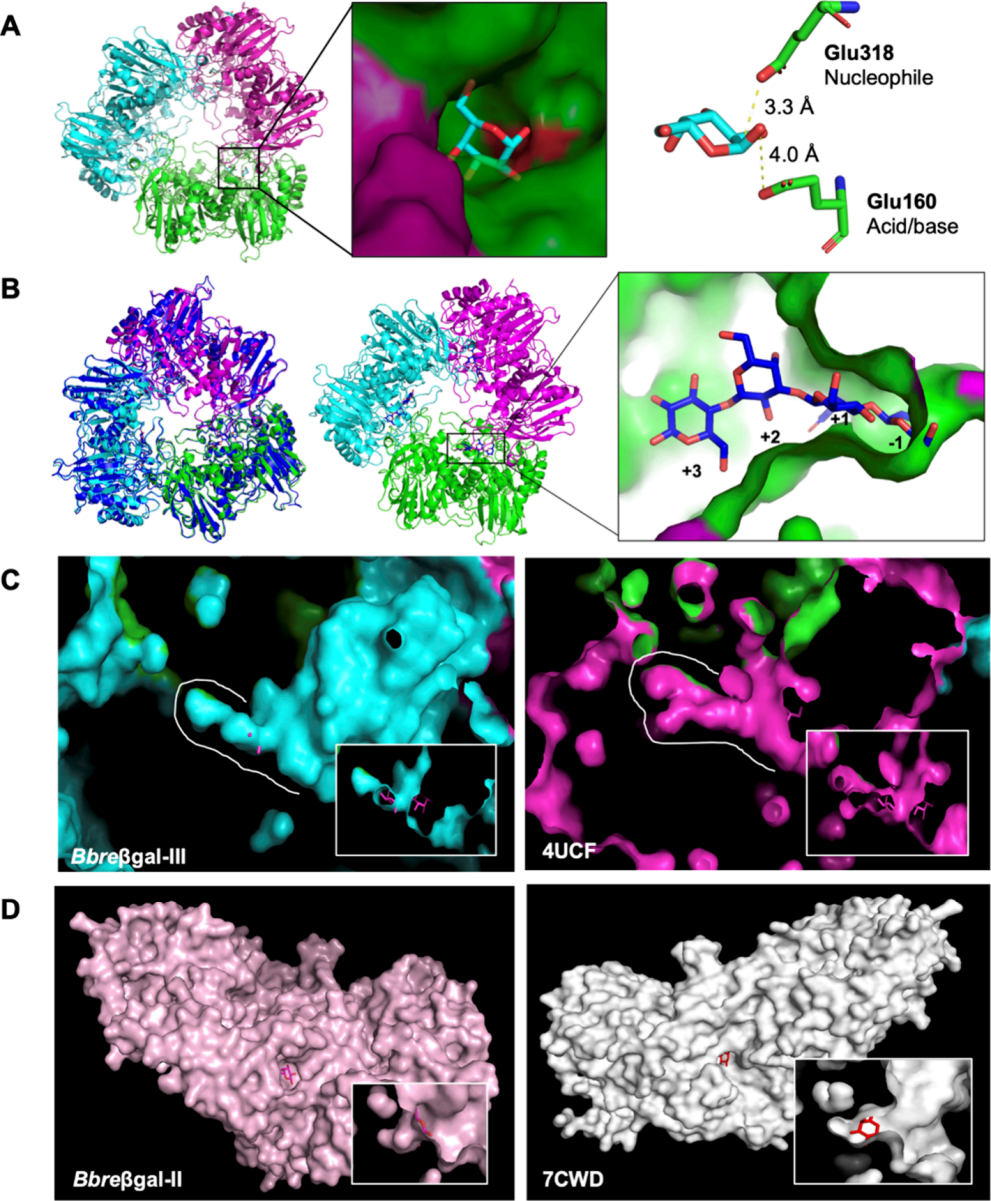
Structure and active site prediction of *Bbre*βgal-III and *Bbre*βgal-II from *B. breve* DSM 20213. (A) Model of *B. breve* DSM 20213 *Bbre*βgal-III predicted by AlphaFold (version 2.2.4) overlaid with the crystal structure of *B. bifidum* S17 β-galactosidase BbgII (PDB 4UCF) in complex with α-galactose (left). The narrow active site of *Bbre*βgal-III is located between the surfaces of adjacent subunits (middle). Docking of α-galactose (cyan pyranose ring) into the active site of *Bbre*βgal-III indicating Glu160 and Glu318 as the acid/base and nucleophile residues, respectively (right). The distance between C1 of the galactose moiety and the nucleophile (Glu318) is 3.3 Å, while the hydroxyl group at C1 is 4 Å from the acid/base residue (Glu160). (B) Model of *B. breve* DSM 20213 *Bbre*βgal-III predicted by AlphaFold (version 2.2.4) overlaid with the crystal structure of *B. longum* subsp. *infantis* β-galactosidase BiBga42A (PDB 8IBT) in complex with lacto-*N*-tetraose (LNT) (left). Crystal structure of BiBga42A (PDB 8IBT) in complex with lacto-*N*-tetraose (LNT). Glucose, galactose (in the lactose moiety, β-d-Gal-(1→4)-d-Glc), and *N*-acetylglucosamine (in the lacto-*N*-biose moiety, LNB, β-d-Gal-(1→3)-β-d-GlcNAc) of LNT occupy the +3, +2, and +1 subsites, respectively. The galactose in the LNB moiety of LNT is in the −1 subsite (right). (C) Deep tunnel-like active sites of bifidobacterial GH42 β-galactosidases: predicted model of *B. breve* DSM 20213 *Bbre*βgal-III (left) and *B. bifidum* S17 BbgII (PDB 4UCF) (right). The white outline indicates the outer surface of the active site. The inset shows a cutaway view revealing how α-galactose fits within the active site. (D) Shallow active sites of GH2 β-galactosidases: predicted model of *B. breve* DSM 20213 *Bbre*βgal-II (left) and *B. circulans* β-galactosidase II (PDB 7CWD) (right). The inset shows a cutaway view, revealing how galactose fits within the active site.

When looking at the active site shapes of several GH42 and GH2 β-galactosidases, it shows that the GH42 enzymes, such as *B. breve* DSM 20213 *Bbre*βgal-III and *B. bifidum* S17 BbgII (PDB 4UCF), possess a deeply buried active site (Figure 5C), whereas the GH2 enzymes, such as *B. breve* DSM 20213 *Bbre*βgal-II and *Bacillus circulans* β-galactosidase II (PDB 7CWD), have a shallow active site (Figure 5D). The difference in the active site architecture clearly affects enzymatic performance, particularly in transgalactosylation, which often requires an optimally shallow active site for easy entry and mobility of the sugar acceptors.^33^

## Discussion

Multiple β-galactosidases are found in several bifidobacteria species, which have unveiled their specific requirements for exerting biological functions as well as their roles in bifidobacterial physiology.^21,34^ We previously reported a study on two GH2 β-galactosidases from *B. breve* DSM 20213, *Bbre*βgal-I and *Bbre*βgal-II.^22^ The third β-galactosidase from *B. breve* DSM 20213, *Bbre*βgal-III, presented in this report belongs to the GH42 family. At the molecular level, *Bbre*βgal-III is distinctively different from the two GH2 β-galactosidases as it is a homotrimer with a molecular weight of 240 kDa, whereas *Bbre*βgal-I and *Bbre*βgal-II are homodimers with molecular weights of 220 and 211 kDa, respectively.

In accordance with β-galactosidases from various sources, *Bbre*βgal-III shows higher preference toward the chromogenic substrate *o*NPG than the natural substrate lactose.^21,22,35–38^ The *K*_m_ values of *Bbre*βgal-III for *o*NPG and lactose, 1.57 and 25.35 mM, respectively, are comparable to reported values of other GH42 β-galactosidases.^12^ The *K*_m_, *k*_cat_, and *k*_cat_/*K*_m_ values of *Bbre*βgal-III for lactose are also comparable to the kinetic parameters of a GH42 β-galactosidase Bga42A (Blon_2016) from *B. longum* subsp. *infantis* ATCC15697, a homologue of *Bbre*βgal-III sharing 96% sequence identity, which were reported to be 16 mM, 97 s^–1^, and 6 mM^–1^ s^–1^, respectively.^34^ The *K*_m_ value determined for lactose of *Bbre*βgal-III, however, was higher than those of *Bbre*βgal-I (15.3 mM) and *Bbre*βgal-II (7.5 mM), which indicates that higher or complete hydrolysis of lactose could be easier to obtain with the two GH2 β-galactosidases than with the GH42 enzyme from *B. breve.*

The optimum pH of *Bbre*βgal-III activity appears to be in the slightly acidic range (pH 5.0 for *o*NPG and pH 5.0–6.5 for lactose hydrolysis), whereas *Bbre*βgal-I and *Bbre*βgal-II show optimal pH values at pH 7.0 and pH 6.5, respectively, for both *o*NPG and lactose.^22^ Furthermore, *Bbre*βgal-III retained more than 75% of its maximal activity for lactose within a broader pH range from 3.5 to 7.0 than *Bbre*βgal-I and *Bbre*βgal-II (pH 6-8). The stability of both enzymes *Bbre*βgal-I and *Bbre*βgal-II rapidly drops at pH values below 6.0 or above 8.0,^22^ while *Bbre*βgal-III demonstrates excellent stability over a broad pH range and is most stable at 5.0. This property might contribute to the ability of these enzymes to degrade HMOs as *B. breve* DSM 20213 is an infant gut isolate. Infant-associated *Bifidobacterium* species degrade HMOs and utilize them as growth substrates. The infant gut typically has a pH around 5.0–6.5, and during bacterial fermentation, the pH in the gut is at the lower end (pH 5.0–5.5), at which only *Bbre*βgal-III is active. It was reported that the GH2 β-galactosidases of *B. breve* UCC2003 are inactive on type I HMO as they do not display the ability to hydrolyze lacto-*N*-tetraose (LNT) while the GH42 β-galactosidase hydrolyses LNT, releasing galactose and lacto-*N*-triose (LNT2). The two GH2 β-galactosidases of *B. breve* UCC2003 are active on the type II HMO in conjunction with the GH42 β-galactosidase as they could hydrolyze LNnT, releasing galactose and LNT2.^18^ It seems that the GH42 β-galactosidase of *B. breve* UCC2003 is more active than the two GH2 β-galactosidases in the degradation of HMOs. We speculate that the GH42 *Bbre*βgal-III of the infant gut isolate *B. breve* DSM 20213 might be involved actively in degradation of HMOs. We also postulate that besides linkage preference in hydrolyzing HMO structures, pH dependence of activity and pH stability of the β-galactosidases of *B. breve* are of vital importance affecting the ability of these enzymes in degradation of HMOs in the guts of breast-fed infants.

The rate constants or partitioning ratios (*k*_Nu_/*k*_water_) can be determined for different acceptors (termed nucleophiles Nu, Scheme 1) under defined initial velocity conditions, and this parameter is used as a measure for the ability of a certain substance to act as a galactosyl acceptor of β-galactosidases, which in turn allows an estimation of the transgalactosylation products obtained for a known reaction mixture or the suitability of a certain enzyme for the efficient synthesis of GOS.^28^ We have reported these *k*_Lac_/*k*_water_ ratios measured for *Bbre*βgal-I and *Bbre*βgal-II, which were 1.61 and 0.53 M^–1^, respectively, under defined initial velocity conditions when the only possible galactosyl acceptors are lactose and its hydrolysis products, d-Gal and d-Glc. Interestingly, the *k*_Lac_/*k*_water_ ratio of *Bbre*βgal-III was determined to be 1.84 M^–1^ (data not shown) indicating higher transferase activity of *Bbre*βgal-III. At the beginning of the reaction, lactose was the preferred galactosyl acceptor during transgalactosylation of lactose catalyzed by *Bbre*βgal-III and the predominant products obtained at ∼40% lactose conversion were the two galactosyllactoses, β-d-Gal*p*-(1→6)-d-Lac and β-d-Gal*p*-(1→3)-d-Lac, accounting for approximately 56 and 13% of the total GOS formed, respectively. On the other hand, d-Glc is a far better galactosyl acceptor for *Bbre*βgal-I and *Bbre*βgal-II as disaccharides other than lactose make up a large proportion of GOS mixtures formed with these two enzymes.^28^

In terms of linkage preference, *Bbre*βgal-III shows a distinct substrate preference with individual galactosides in comparison with *Bbre*βgal-I and *Bbre*βgal-II (Figure 2). The GH2 β-galactosidases *Bbre*βgal-I and *Bbre*βgal-II exhibit substrate preferences toward β-d-Gal*p*-(1→4)-d-Glc (lactose), β-d-Gal*p*-(1→6)-d-Glc (allolactose), and β-d-(1→3)-linked di- and trisaccharides. On the other hand, GH42 β-galactosidase *Bbre*βgal-III displays significantly lower affinities toward lactose and allolactose than for β-d-(1→3)-linked di- and trisaccharides, 6′-galactobiose and 6′-galactosyllactose. These typical features align with other GH42 β-galactosidases.^12,21,34^ *Bbre*βgal-III has strong preferences to form β-d-Gal*p*-(1→6)-d-Lac and β-d-Gal*p*-(1→3)-d-Lac but also rapidly hydrolyzes these structures. 3′- and 6′-Galactosyllactoses are the preferred substrates for hydrolysis by *Bbre*βgal-III, even when high amount of lactose is still present in the reaction mixture during the conversion; hence, pronounced degradation of these GOS was already observed at ∼ 40% lactose conversion. It explains why a relatively low GOS yield was obtained with *Bbre*βgal-III. In contrast, higher GOS yields of 33 and 38% of total sugars were obtained with *Bbre*βgal-I at 70% lactose conversion and *Bbre*βgal-II at 96% lactose conversion, respectively,^22^ because the hydrolysis of formed GOS was only pronounced toward the end of the reactions when the substrate lactose became depleted. It is conceivable that β-galactosidases, which can rapidly hydrolyze certain GOS structures, will favorably form these structures when acting in transgalactosylation mode.^25^ 4′-Galactobiose (β-d-Gal*p*-(1→4)-d-Gal) was not detected at all during lactose conversions by all three β-galactosidases *Bbre*βgal-I, *Bbre*βgal-II, and *Bbre*βgal-III, which is in agreement with our observation that this disaccharide is not a preferred hydrolytic substrate for these enzymes (Figure 2).

Furthermore, *Bbre*βgal-III shows high preference for all three 3′-, 4′-, and 6′-galactosyllactoses, while *Bbre*βgal-I and *Bbre*βgal-II have lower preference toward 4′- and 6′-galactosyllactoses in comparison with lactose. These findings suggest that multiple β-galactosidases in *B. breve* play distinctive roles in the utilization of a diverse range of substrates under different conditions, especially in the assimilation of various HMO structures containing one or more β-linked galactose moieties as selective substrates for the growth and well-being of breast-fed infants. Additionally, the GH42 β-galactosidase *Bi*Bga42A from *B. longum* subsp. *infantis* ATCC15697 (PDB 8IBT), a homologue of *Bbre*βgal-III (96% identity), has been shown to selectively degrade type I HMOs (lacto-*N*-tetraose, LNT),^31,34^ which contains β-1,3-linked galactose. Gotoh et al. discussed the structural basis underlying LNT hydrolysis by *Bi*Bga42A and the active site of *Bi*Bga42A (PDB 8IBT) with bound LNT, with the LNB (β-d-Gal-(1→3)-β-d-GlcNAc) disaccharide structure bound in subsites −1 and +1 and the lactose (β-d-Gal-(1→4)-d-Glc) disaccharide structure in subsites +2 and +3 (Figure 5B).^31^ Structural alignment showed that our predicted model of *Bbre*βgal-III agrees with the experimentally solved crystal structure of *Bi*Bga42A (Figure 5B). This suggests that *Bbre*βgal-III might also play a role in the degradation of type I HMOs.

Unlike *B. bifidum* S17 BbgII (PDB 4UCF), which lacks the ability to synthesize GOS, *Bbre*βgal-III shows a noteworthy difference in our present study. GOS formation was observed with *Bbre*βgal-III, yielding a maximum of 17% of total sugars (at ∼70% lactose conversion). BgaB from *B. breve* UCC2003 and BgaG (also known as Bga42A or Blon_2016 described in ref 34) from *B. longum* subsp. *infantis* ATCC15697 sharing 99 and 96% sequence identity with *Bbre*βgal-III showed comparable GOS yields of 18 and 14%, respectively.^11^ In comparison with the GH2 β-galactosidases from the same strain, both *Bbre*βgal-I and *Bbre*βgal-II displayed significantly higher GOS yields, which are 33 and 38% of total sugars, respectively. This suggests that the GOS synthesizing activity is influenced by the catalytic residues and their interactions with other residues in the active site or in their vicinity as well as the location and shape of the active sites. The active site of the GH42 BbgII from *B. bifidum* S17 (PDB 4UCF) is located deep inside the barrel of one domain,^12^ while the active sites of the GH2 β-galactosidase from *B. circulans* (PDB 7CWD) and our predicted GH2 *Bbre*βgal-II from *B. breve* DSM 20213 are relatively surface-exposed (Figure 5B,C). The architecture of a surface-exposed active site might allow GH2 β-galactosidases to interact more rapidly with the substrates or galactosyl acceptors compared to GH42 enzymes with buried active sites as the substrates must pass through the channel prior to binding at the active sites.^39^ Therefore, we argue that the active site cavity may contribute to the fact that GH42 β-galactosidases have only weak or complete lack of GOS synthesis abilities.

In conclusion, the GH42 β-galactosidase from *B. breve* DSM 20213, *Bbre*βgal-III, was extensively investigated regarding different aspects of its biochemical and molecular properties, including its substrate preferences and its ability to catalyze transgalactosylation reactions. Despite giving lower GOS yields when compared to *Bbre*βgal-I and *Bbre*βgal-II and other GH2 β-galactosidases, *Bbre*βgal-III has significant potential for prebiotic GOS production, especially when it comes to the formation of certain specific GOS structures such as 6′ and 3′-galactosyllactoses. This preference suggests that *Bbre*βgal-III is a potential candidate for the synthesis of GOS mixtures containing specific components to produce functionally enhanced prebiotics. However, further improvement on the yields of these formed structures is necessary, and this can be corroborated by enzyme engineering, which is ongoing in our laboratory.

## Supplementary Material



## Notes

(Table S1) Operating conditions of the UPLC-Orbitrap MS for analysis of polysaccharides; (Table S2) pH stability of recombinant β-galactosidase *Bbre*βgal-III incubated at 37 °C in Britton-Robinson buffer; (Figure S1) SDS-PAGE analyses of crude cell-free extracts of *E. coli* BL21 Star (DE3) and purified *Bbre*βgal-III; (Figure S2) Spectra from size-exclusion chromatography for molecular weight determination of recombinant β-galactosidase *Bbre*βgal-III; (Figure S3) Thermostabilty of recombinant β-galactosidase *Bbre*βgal-III in 50 mM sodium phosphate buffer, pH 6.5 (PDF)
