# Supplementary material for: A GH42 β‑Galactosidase from the Human Isolate Bifidobacterium breve DSM 20213: Biochemical and Transgalactosylation Properties Reveal the Potential for Galacto-oligosaccharides Synthesis
Source: ACS Omega. 2025 Dec 17;10(51):62633–44. doi: 10.1021/acsomega.5c06811 (PMC12756740; doi:10.1021/acsomega.5c06811)
Supplement: Supplementary file 1 [file ao5c06811_si_001.pdf]

A GH42 β-galactosidase from the human isolate *Bifidobacterium breve* DSM 20213: biochemical and transgalactosylation properties reveal the potential for galacto-oligosaccharides synthesis

Khanh-Trang Vu-Le[1,2], Konlarat Phirom-on[1,3], Sineenat Sripattanakul[4,5], Dinh Binh Chu[6], Stephan Hann[7], Markus Blaukopf[8], Leander Suetzl[1], Chris Oostenbrink[3,9], Dietmar Haltrich[1,3], Thu-Ha Nguyen[1*]

[1]Food Biotechnology Laboratory, Department of Biotechnology and Food Science, BOKU University, Muthgasse 18, A-1190 Vienna, Austria

[2]Faculty of Biology and Environmental Science, The University of Danang - University of Science and Education, Danang, Vietnam

[3]Doctoral Programme BioToP - Biomolecular Technology of Proteins, BOKU University, Muthgasse 18, A-1190 Vienna, Austria

[4]Office of Research Administration, Chiang Mai University, Chiang Mai 50200, Thailand

[5]Laboratory of BioMolecular Imaging, Molecular and Cellular Biology, Department of Radiologic Technology, Faculty of Associated Medical Sciences, Chiang Mai University, Chiang Mai 50200, Thailand

[6]Faculty of Chemistry, School of Chemistry and Life Sciences, Hanoi University of Science and Technology, 1 Dai Co Viet, Hanoi, Vietnam

[7]Institute of Analytical Chemistry, Department of Natural Sciences and Sustainable Resources, BOKU University, Muthgasse 18, A-1190 Vienna, Austria

[8]Institute of Organic Chemistry, Department of Natural Sciences and Sustainable Resources, BOKU University, Muthgasse 18, A-1190 Vienna, Austria

[9]Institute of Molecular Modeling and Simulation, Department of Natural Sciences and Sustainable Resources, BOKU University, Muthgasse 18, A-1190 Vienna, Austria

[*] Correspondence: thu-ha.nguyen@boku.ac.at

Supporting information

Table S1. Operating conditions of UPLC-Orbitrap MS for analysis of polysaccharides

**UPLC operating conditions**	
UPLC system	Vanquish Horizon UPLC System
Stationary phase	Supelco Supel™ Carbon LC Guard Cartridge (20×2.1 mm, 2.7 µm)
Mobile phase	Acetonitrile (ACN) and water containing 0.1% formic acid (FA)
Flow rate of mobile phase	0.3 mL min^{-1}
Elution profile (gradient of mobile phase)	Chanel A: 0.1% FA in deionized water<br>Chanel B: 0.1% FA 95% ACN and 5% deionized water
Injection volume	5 µL
Column temperature	30 $^{\circ}$C
Sample chamber temperature	10 $^{\circ}$C
**HESI Orbitrap MS operating operations**	
Ionization sources	Heated electrospray ionization (HESI)
Temperature	300 $^{\circ}$C
Potential voltages	+ 3.5 kV in positive mode
Sheath gas	32 psi
Auxiliary gas	7 L min^{-1}
**Orbitrap MS operating conditions**	
Resolution of mass spectrometry	30000 for full MS and 15000 for dd-MS/MS
Measurement mode	Full MS and dd-MS/MS
Mass range	120-1200 m/z for full scan
Data format	Profile for full MS
Data format	Centroid for dd-MS^2
Collision energy	30% NCE
Isolation mass window	1.2 m/z for dd-MS^2

Table S2. pH stability of recombinant β-galactosidase *Bbre*βgal-III incubated at 37 °C in Britton-Robinson buffer

**pH**	3.0	3.5	4.0	4.5	5.0	5.5	6.0	6.5	7.0	7.5	8.0	8.5	9.0
**Half-life of activity ($\tau_{1/2}$)**	7 h	8 h	30 h	72 h	67 days	60 days	60 days	45 days	30 days	30 days	22 days	7 days	24 h

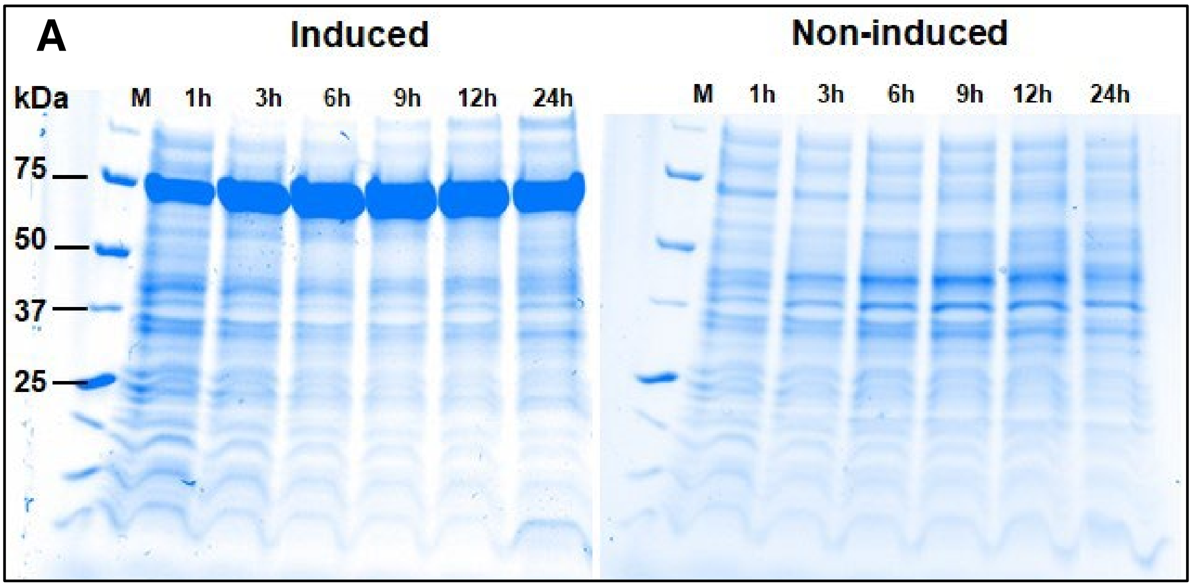

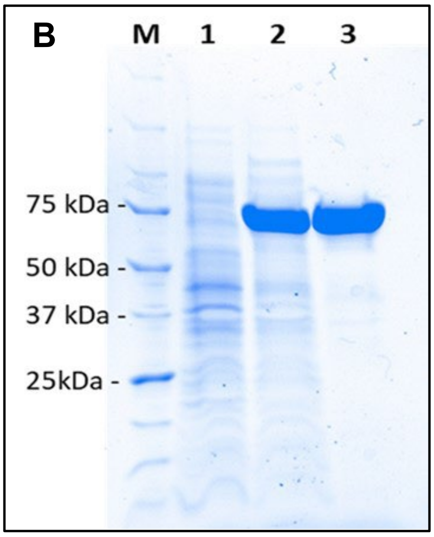

Figure S1. SDS-PAGE analyses

(A) Crude cell-free extracts of *E. coli* BL21 Star (DE3). *E. coli* BL21 Star (DE3) carrying the plasmid pET21a-Bbreβgal-III was cultivated at 37 °C in 300 mL of LB broth supplemented with 100 µg mL^{-1} ampicillin until reaching an OD_{600} of 0.6. The cultures were induced with 0.1 mM IPTG (see Materials and Methods for details). The samples were harvested at different time points after induction. M denotes the Precision protein ladder (Biorad, CA, USA).

(B) Purified *Bbre*βgal-III. M denotes the Precision protein ladder (Biorad, CA, USA); 1, 2, 3 denotes: non-induced crude enzyme, induced crude enzyme, and purified enzyme, respectively.

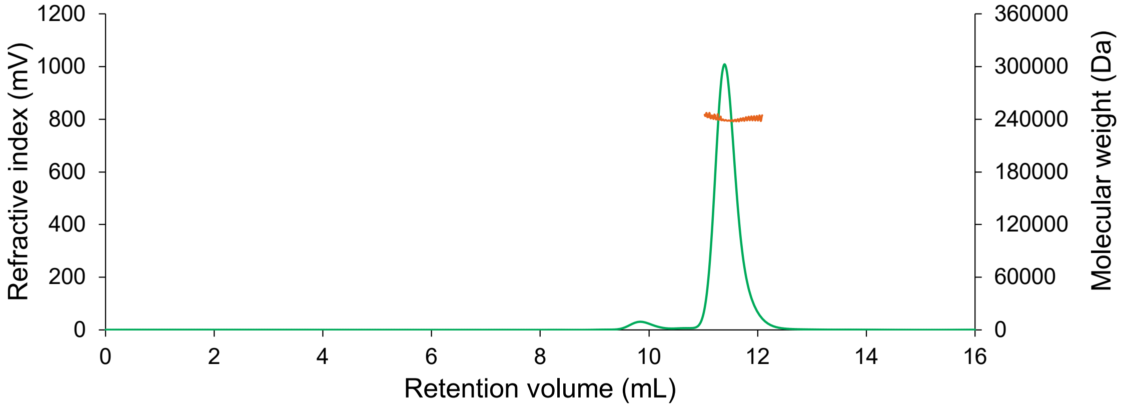

Figure S2. Spectra from size exclusion chromatography with an integrated triple-detector system (Refractive Index, Light Scattering and UV/VIS PDA) for molecular weight determination of *Bbre*βgal-III. The enzyme shows one main peak with a molecular weight of approximately 240 kDa.

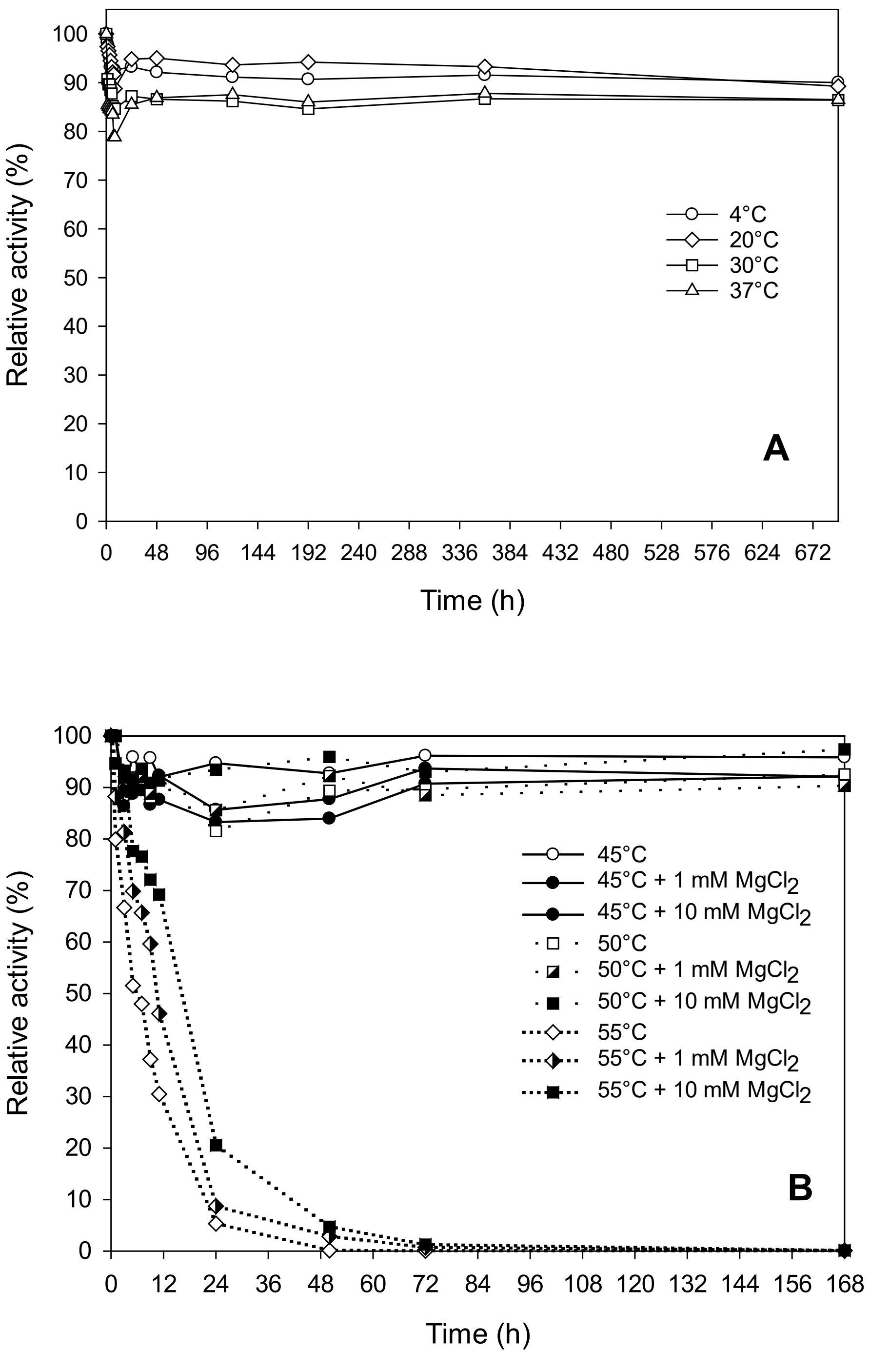

Figure S3. Thermostabilty of recombinant β-galactosidase *Bbre*βgal-III in 50 mM sodium phosphate buffer, pH 6.5

(A) Lower temperature range (4 - 37°C)

(B) Elevated temperature range (45 - 55°C) without or in the presence of $MgCl_2$. The first time point t = 0 h corresponds to immediate measurement of enzyme activity after mixing with $MgCl_2$.
